# Acute-onset severe gastrointestinal tract hemorrhage in a postoperative patient taking rivaroxaban after total hip arthroplasty: a case report

**DOI:** 10.1186/1752-1947-6-129

**Published:** 2012-05-14

**Authors:** Michael Boland, Maria Murphy, Marese Murphy, Enda McDermott

**Affiliations:** 1 Department of Surgery, St Vincent’s University Hospital, Elm Park, Dublin 4, Ireland

## Abstract

**Introduction:**

Rivaroxaban, a new oral anticoagulant, is currently licensed for use in patients undergoing orthopedic surgery. It is more efficacious than other anticoagulants such as low molecular weight heparin and does not require daily monitoring. It has also been shown to be efficacious in patients with venous thromboembolism and acute coronary syndrome. Although hemorrhage is a known side effect of this new anticoagulant, we could find no case reports in the literature of patients suffering severe hemorrhage whilst taking rivaroxaban. Thus, we describe the first case of potentially fatal hemorrhage in a patient taking rivaroxaban.

**Case presentation:**

We report the case of a 58-year-old Caucasian man with acute-onset severe per rectal bleeding who had undergone total hip arthroplasty four weeks prior to the onset of symptoms and was taking rivaroxaban in the postoperative period. Rivaroxaban was discontinued immediately but, having required nine units of packed red blood cells in a peripheral hospital due to a rapidly decreasing hemoglobin level, our patient was transferred to our tertiary referral center where he required a further eight units of packed red blood cells over a 48-hour period to manage his ongoing hemorrhage and maintain hemodynamic stability. No source of bleeding was found on computed tomography angiography and our patient’s condition improved over the following 48 hours with cessation of the hemorrhage. Our patient was discharged home well several days later. A follow-up colonoscopy one week after his discharge was normal.

**Conclusion:**

Although advantageous with regard to its oral availability and ongoing use without the need for daily monitoring, rivaroxaban does not come without rare but severe side effects. When severe per rectal bleeding occurs in a patient taking rivaroxaban, discontinuation of the offending agent and aggressive hematological replacement are the mainstays of treatment, especially when no source of bleeding can be found. This case, as the first to describe severe hemorrhage and rivaroxaban, serves as a reminder to those prescribing the medicine that they must inform the patient of the risk of such a serious side effect and the need for urgent medical attention if it occurs.

## Introduction

The new anticoagulant rivaroxaban, introduced to the Irish Health System over three years ago, was the first in a new line of exciting oral anticoagulant agents. Working by inhibiting Factor Xa, rivaroxaban stops the formation of thrombin as the extrinsic and intrinsic pathways of the coagulation cascade converge. As a therapeutic agent, it boasts good pharmacokinetic qualities but the outstanding advantage over competing anticoagulants is that there is no need for routine coagulation monitoring and subsequent dose adjustments. Currently, rivaroxaban is used clinically after total hip arthroplasty and knee replacement surgery. The recent RECORD 1–4 Trials found that a once daily oral dose of rivaroxaban was more effective for extended prophylaxis than a once daily dose of subcutaneous enoxaparin in patients undergoing the surgeries mentioned above, with the two drugs having similar safety profiles. However, the main side effect associated with this new drug remains severe hemorrhage.

We describe a severe per rectum (PR) bleed in our patient who had undergone total hip arthroplasty (THA) four weeks prior to the onset of symptoms and had been taking rivaroxaban postoperatively. We focus on the rare but very serious side effect of major bleeding in a patient taking rivaroxaban, the management of this side effect and the considerations that need to be taken when starting a patient on this new anticoagulant. Most notably, no previous cases of severe hemorrhage associated with the use of rivaroxaban could be found in the literature and so we describe this side serious effect in a case report for the first time.

## Case presentation

The case involves a 58-year-old Caucasian man who presented to a regional hospital with a 24-hour history of severe PR bleeding. Our patient described the bleeding as being acute in onset, bright red with clots and associated with mild left iliac fossa pain. He had no other gastrointestinal symptoms prior to the bleed and had no history of colorectal disease. Of note, he had undergone a THA 31 days prior to his transfer from the regional hospital to our tertiary referral center and was taking oral rivaroxaban as a prophylactic anticoagulant in the postoperative period at a dose of 10 mg once daily. He was on no other medications and had no history of renal disease.

On arrival at the regional center, our patient had hemodynamic stability but his PR bleeding continued with multiple episodes involving an average of 250 to 500 mL in volume. His hemoglobin (Hb) level dropped from 12.0 g/dL on admission to 10.3 g/dL and subsequently to 7.4 g/dL over a 24-hour period. His coagulation profile and platelet levels were normal as was his renal function. An abdominal computed tomography scan revealed uncomplicated diverticular disease but no other abnormalities. Upper gastrointestinal endoscopy was also performed, which showed very mild gastritis but no source of hemorrhage. In the 24 hours between admission and transfer to our tertiary referral center, he received a total of nine units of packed red blood cells (PRBCs) in order to maintain hemodynamic stability.

Following transfer to our tertiary referral center, our patient had ongoing bleeding but remained hemodynamically stable. His vitals were as follows: heart rate, 98 beats/minute; blood pressure (BP), 134/71 mmHg; respiratory rate, 20 breaths/minute; oxygen saturation level, 98% on room air; and temperature, 36.0°C. On examination, he was pale but alert and chatty. His abdomen was soft with some mild tenderness in the left iliac fossa and there were no palpable masses, no evidence of organomegaly and bowel sounds were present. A PR examination was positive for fresh blood. His respiratory and cardiovascular examinations were both normal. His Hb level on admission was 8.3 g/dl (as shown in Table 1). Our patient was kept strictly nil by mouth and commenced on eight hourly intravenous fluids. A group and cross-match was taken with four units of PRBCs on standby. He was to have repeat Hb levels taken if further hemorrhage occurred and his hemodynamic parameters were observed every two hours. After a further bleed overnight, he received one unit of PRBCs.

**Table 1:** Hemoglobin values and packed red blood cells given on admission and at intervals after admission to our center

**Time from admission** **(hours)**	**Hemoglobin level** **(g/dL)**	**Units of packed red blood cells given**
0	8.3	1
8	9.0	3
16	8.5	2
20	7.6	2
24	10.4	-
32	11.4	-
44	9.4	-
54	8.9	-
60	10	-

During the next 15 hours, our patient had a further two episodes of fresh blood PR whilst receiving three units of PRBCs. After the second episode his Hb level was 8.5 g/dL and he was prescribed a further two units of PRBCs as well as being commenced on an intravenous proton pump inhibitor. His BP subsequently dropped to 90/57 mmHg and his intravenous fluid regime was increased to 1 L every four hours with orders that he also receive Gelofusine (a succinylated gelatin solution) if his BP dropped again. He had four further episodes of bleeding over the following six hours and received two more units of PRBCs during this period. This was a total of eight units since his arrival to our tertiary referral center 24 hours earlier and 17 units in total since his presentation.

Due to the increasing frequency of these hemorrhagic episodes, our patient underwent emergency computed tomography mesenteric angiography. A consultant interventional radiologist could identify no obvious source of bleeding but ordered that our patient be returned immediately to the interventional radiology suite for embolization if further episodes of fresh blood PR occurred. His Hb level at this time was 11.4 g/dL and our patient was hemodynamically stable.

Over the next four days, our patient improved significantly with no further episodes of PR bleeding. He resumed a normal diet after three days and was discharged home well with a Hb level on discharge of 10.4 g/dL. His renal function remained within normal limits throughout his hospital stay.

Our patient returned six days later, having experienced no further symptoms, and underwent a colonoscopy that was entirely normal. When he was seen again six weeks later in the outpatient clinic; he was asymptomatic and reported feeling great. He was subsequently discharged from our service.

## Discussion

Rivaroxaban is an oral anticoagulant that works by directly inhibiting Factor Xa and so interrupting both the intrinsic and extrinsic pathway of the coagulation cascade and subsequent thrombus formation. It is the first orally active direct Factor Xa inhibitor and was granted marketing authorization in 2008 by both the European Commission and Health Canada for the prevention of venous thromboembolism in patients who have undergone elective total hip replacement and total knee replacement surgery. Rivaroxaban’s authorization comes as a result of the recent RECORD 1–4 Trials, which looked at its efficacy versus subcutaneous enoxaparin as a thromboprophylaxis in patients undergoing THA and total knee arthroplasty [1-4]. In the RECORD 1 trial, a randomized double-blinded study, 4,518 patients were assigned to receive 10 mg daily of oral rivaroxaban immediately after THA or 40 mg once daily of subcutaneous heparin beginning the same evening after THA [1]. The primary efficacy outcome was the composite of deep venous thrombosis, nonfatal pulmonary embolism or death after 36 days and, significantly, the major safety outcome was major bleeding. The primary efficacy outcome occurred in 18 of 1,595 patients (1.1%) in the rivaroxaban group and in 58 of 1,558 patients (3.7%) in the enoxaparin group (absolute risk reduction, 2.6%; 95% confidence interval, 1.5 to 3.7; P < 0.001). Major bleeding occurred in 6 of 2,209 patients (0.3%) in the rivaroxaban group and in 2 of 2,224 patients (0.1%) in the enoxaparin group (P = 0.18). The authors concluded that a once daily oral dose of rivaroxaban was more effective for extended thromboprophylaxis than a once daily subcutaneous dose of enoxaparin in patients undergoing elective THA, with the two drugs having similar safety profiles. The manufacturers recommend that rivaroxaban only needs be taken for 35 days postoperatively, after which the risk of such a thrombotic event decreases hugely. Although six cases of major bleeding were reported in the RECORD 1 trial, we could find no available case reports in the literature regarding this side effect.

The circumstances of our patient’s case demonstrated very clearly both the obvious advantages and yet the severe disadvantages of rivaroxaban. Upon its formulation, the immediate appeal of this drug was three-fold. First, it was more efficacious in preventing deep venous thrombosis or pulmonary embolisms compared to other anticoagulants, as shown in the RECORD trials. Second, it could be taken orally, negating the need for daily subcutaneous injections that patients experience if taking heparin or a low-molecular-weight heparin, most notably in the immediate days after surgery. Third, and perhaps most significantly from a socioeconomic perspective, regular visits to general practitioners or outpatient clinics for international normalized ratio monitoring when taking warfarin are also unnecessary when taking rivaroxaban. Interestingly, rivaroxaban is the first oral Factor Xa inhibitor licensed for this purpose in Ireland. It will most likely set a trend for a class of oral anticoagulants that will see the end of subcutaneous anticoagulation as well as oral anticoagulants that require continuous blood level monitoring. Although only currently licensed for orthopedic surgery, more recent studies have shown that rivaroxaban is as effective as enoxaparin when treating symptomatic venous thromboembolism. In addition, a Phase III study of low-dose rivaroxaban in patients with acute coronary syndrome is also underway after a recent Phase II study showed that it may reduce major ischemic events [5,6].

In this case, the fact that our patient had no history of hemorrhage in the past and that the organ and associated system involved, the rectum and gastrointestinal tract, had never caused him medical problems prior to this makes the magnitude of the PR bleeding more noteworthy. The absence of any risk factors would have placed him in a low-risk category for side effects. The RECORD 1 trial found reports of major bleeds in only six patients and this case demonstrates that these side effects, though rare, do occur in supposedly low-risk patients, with serious and potentially fatal complications.

The treatment of such a severe side effect includes transfusion with PRBCs based on regularly observed Hb levels. One could consider the role of radiological embolization if a source is found but this was not an option in our case. The most important learning point, however, is that the offending agent is discontinued as quickly as possible. This relies on a detailed history with specific focus on recent pharmacological treatments. Rivaroxaban has a mean terminal half-life of seven to eleven hours, which should be taken into account along with other medications that the patient is taking, such as antimycotics or human immunodeficiency virus protease inhibitors, which can potentiate bleeding. Once the agent is discontinued, bridging the patient through the period of hemorrhage safely is the primary goal. The lack of any obvious antidote to reverse the effects of the drug is an aspect that needs further research. Early studies have shown that prothrombin complex concentrate may be useful in reversing the effects of rivaroxaban [7]. Other possible measures include the use of recombinant Factor VIIa to reduce bleeding or the use of activated charcoal to reduce absorption in cases of overdose.

## Conclusion

This case outlines clearly, and for the first time in the literature, the rare but serious risk of hemorrhage associated with rivaroxaban. Prescribing orthopedic specialists should take care in providing information regarding the risk of bleeding to patients commencing the medication and inform these patients that urgent medical attention is needed if such a side effect occurs. The efficacy and ease of use associated with this new anticoagulant means that its use is likely to increase across multiple medical specialties, making this case important for a wide range of medical professionals. This case also outlines how such a side effect is managed effectively and that immediate discontinuation of rivaroxaban is a vital step in dealing with hemorrhage. This case has not altered the use of rivaroxaban in our health facility but has drawn attention to a serious side effect of the drug.

## Consent

Written informed consent was obtained from the patient for publication of this case report and any accompanying images. A copy of the written consent is available for review by the Editor-in-Chief of this journal.

## Abbreviations

BP: blood pressure; Hb: hemoglobin; PR: per rectum; PRBCs: packed red blood cells; THA: total hip arthroplasty.

## Competing interests

The authors declare that they have no competing interests and received no funding for the production of this case report.

## Authors’ contributions

MB drafted and critically revised the manuscript. MM contributed to drafting the article and collected data. MM contributed to drafting and critically revised the manuscript. EMcD contributed to the drafting of the manuscript. All authors read and approved the final manuscript.

## References

[B1] Eriksson BI Borris LC Friedman RJ Haas S Huisman MV Kakkar AK Bandel TJ Beckmann H Muehlhofer E Misselwitz F Geerts W RECORD1 Study Group Rivaroxaban versus enoxaparin for thromboprophylaxis after hip arthroplasty N Engl J Med 2008 358 26 2765 2775 10.1056/NEJMoa0800374 18579811

[B2] Kakkar AK Brenner B Dahl OE Eriksson BI Mouret P Muntz J Soglian AG Pap AF Misselwitz F Haas S RECORD2 Investigators Extended duration rivaroxaban versus short-term enoxaparin for the prevention of venous thromboembolism after total hip arthroplasty: a double blind randomised controlled trial Lancet 2008 372 9632 31 39 10.1016/S0140-6736(08)60880-6 18582928

[B3] Lassen MR Ageno W Borris LC Lieberman JR Rosencher N Bandel TJ Misselwitz F Turpie AG RECORD3 Investigators Rivaroxaban versus enoxaparin for thromboprophylaxis after total knee arthroplasty N Engl J Med 2008 358 26 2776 2786 10.1056/NEJMoa076016 18579812

[B4] Turpie AG Lassen MR Davidson BL Bauer KA Gent M Kwong LM Cushner FD Lotke PA Berkowitz SD Bandel TJ Benson A Misselwitz F Fisher WD RECORD4 Investigators Rivaroxaban versus enoxaparin for thromboprophylaxis after total knee arthroplasty (RECORD4): a randomised trial Lancet 2009 373 9676 1673 1680 10.1016/S0140-6736(09)60734-0 19411100

[B5] Bauersachs R Berkowitz SD Brenner B Buller HR Decousus H Gallus AS Lensing AW Misselwitz F Prins MH Raskob GE Segers A Verhamme P Wells P Agnelli G Bounameaux H Cohen A Davidson BL Piovella F Schellong S EINSTEIN Investigators Oral rivaroxaban for symptomatic venous thromboembolism N Engl J Med 2010 363 26 2499 2510 21128814 10.1056/NEJMoa1007903

[B6] Mega JL Braunwald E Mohanavelu S Burton P Poulter R Misselwitz F Hricak V Barnathan ES Bordes P Witkowski A Markov V Oppenheimer L Gibson CM ATLAS ACS-TIMI 46 study group Rivaroxaban versus placebo in patients with acute coronary syndromes (ATLAS ACS-TIMI 46): a randomised, double-blind, phase II trial Lancet 2009 4 9683 29 38 10.1016/S0140-6736(09)60738-8 19539361

[B7] Eerenberg ES Kamphuisen PW Sijpkens MK Meijers JC Buller HR Levi M Reversal of rivaroxaban and dabigatran by prothrombin complex concentrate: a randomized, placebo-controlled, crossover study in healthy subjects Circulation 2011 124 14 1573 1579 10.1161/CIRCULATIONAHA.111.029017 21900088

